# Association of activity-based food environment index with obesity-related cancer mortality in the US

**DOI:** 10.1186/s12916-025-03967-6

**Published:** 2025-03-20

**Authors:** Qinyun Lin, Xiang Chen, Xukun Xiang, Weixuan Lyu, Congcong Miao, Gaofei Zhang, Ran Xu

**Affiliations:** 1 School of Public Health and Community Medicine, Institute of Medicine, University of Gothenburg, Guldhedsgatan 5A, Plan 3, Gothenburg, Sweden; 2 Department of Geography, Sustainability, Community and Urban Studies, University of Connecticut, Storrs, USA; 3 Independent Researcher University of Chicago, Chicago, USA; 4 Department of Allied Health Sciences, College of Agriculture, Health and Natural Resources, University of Connecticut, Storrs, USA

**Keywords:** Retail food environment, Obesity-related cancer mortality, Activity-based index, Location-based index, Human mobility

## Abstract

**Background:**

Obesity and obesity-related cancers contribute to rising healthcare costs and declining life expectancy in the US and improving diet quality plays a crucial role in reversing such trends. Existing studies on the relationship between healthy food access and obesity-related cancer mortality present mixed findings, whereas food procurement activities are largely overlooked. The paper aims to construct a novel food environment index based on residents’ food retailer visits, and then compare it with the location-based food environment index regarding the strength of associations with obesity-related cancer mortality rates.

**Methods:**

This cross-sectional ecologic study used business location data from InfoGroup and aggregated GPS-based food retailer visit data from SafeGraph in 2018–2019, and mortality data from the Centers for Disease Control and Prevention in 2015–2020. A total of 2925 counties or equivalents with complete information were included. Activity-based index was calculated as the percentage of visits to healthy food retailers out of total visits to all qualified food retailers for residents in each county. Location-based index was calculated as the percentage of healthy food retailers out of all qualified food retailers in each county. The main outcome is age-adjusted obesity-related cancer (13 types of cancer based on evidence from the International Agency for Research on Cancer) mortality rates, which were calculated for each county and counties were further categorized into high- and low-risk (≥ 60.2 and < 60.2 cases per 100,000 population) areas. Linear, non-linear, logistic, and spatial regression analyses were performed to examine the association between each food environment index and obesity-related cancer mortality rates.

**Results:**

The activity-based index demonstrated significant negative association with the 2015–2020 obesity-related cancer mortality rates (coefficient [95% CI]: − 0.980 [− 1.385, − 0.575], *P* < 0.001), and each standard deviation increase in the activity-based index was associated with an 18% decrease in the odds of being in a high-risk area (odds ratio [95% CI]: 0.821 [0.749, 0.900], *P* < 0.001), while the location-based index showed much weaker and non-significant effects.

**Conclusions:**

Our findings suggest that health policies and initiatives that combat obesity and obesity-related cancers should consider incorporating food retailer visits into policy formation.

**Supplementary Information:**

The online version contains supplementary material available at 10.1186/s12916-025-03967-6.

## Background

Obesity and obesity-related cancers are prevalent and are major drivers of rising healthcare costs, diminished health-related quality of life, and the recent decline in life expectancy in the US [1]. The most recent data shows that 41.9% and 73.6% of the adults aged 20 years and older in the US are obese and overweight [2, 3]. It is estimated that about 5% of new cancer cases in men and 10% in women are attributable to excess body weight every year [4], and the International Agency for Research on Cancer (IARC) has linked excess adiposity to 13 types of cancers, including esophageal cancer, gastric cardia cancer, colorectal cancer, liver cancer, gallbladder cancer, pancreatic cancer, laryngeal cancer, postmenopausal breast cancer, endometrial cancer, ovarian cancer, kidney cancer, thyroid cancer, and multiple myeloma [5].

While it is well recognized that diet quality is associated with obesity and obesity-related cancer incidence and mortality [6–12], the environmental determinants of diet quality on the individual level or the community level remain far from conclusive. It is widely recognized that a major environmental determinant is access to healthy/unhealthy food provisioning, which is usually measured by geographical models of spatial access (e.g., proximity, density, and variety) to relative health-promoting retailers (e.g., grocery stores) or health-damaging ones (e.g., fast-food restaurants) in one’s residential neighborhood [13]. Because of the presumed link between food environments and diet quality, many national food and health initiatives have incorporated food environment measures, including the United States Department of Agriculture (USDA) Food Access Research Atlas [14] and the Centers for Disease Control and Prevention (CDC) modified retail food environment index (mRFEI) [15], as evidence in designing policy interventions to improve diet-related health outcomes [16].

However, past literature presents mixed findings on the connection between such food environment measures and obesity or obesity-related cancers. For example, some studies identified a positive relationship between access to healthy/unhealthy foods and diet quality, obesity and obesity-related cancers [17–22], but others found negative [23], null [24], and mixed relationships [25–28]. While many factors could contribute to these inconsistencies, one important but often overlooked aspect is food-related human activity, such as food retailer visits [29]. Specifically, the underlying assumption of establishing these place-health connections is that food consumers are exposed to all food retailers within a predefined administrative unit (e.g., city, county, or census tract) and can alter their health behaviors, including their diets, due to such exclusive exposure. However, in practice, consumers often engage in food procurement activities that occur at multiple locations or span over multiple units [30], and their food exposure and behaviors may not accurately reflect the foodscape surrounding their residence due to various behavioral uncertainties, such as food culture, health education, and food perceptions [31–33]. For example, recent studies have shown that the majority of the food retailer visits occurred outside of residents’ immediate neighborhoods, and therefore, their patterns of food retailer visit differed significantly from those defined by their residential food environment [33, 34].

Several previous studies have linked food retailer visits to diet quality and obesity, mostly on the local scale [35–40]. To our knowledge, there is no study examining the association between food retailer visits and obesity-related cancer mortality on the national level. To fill the gap, this study leverages a large-scale Global Positioning System (GPS)-based human mobility dataset covering over 94 million aggregated visit records to roughly 359,000 food retailers across the US for 2 years. In this study, we have examined a novel index based on county-level food retailer visits and compared it with the widely used location-based food environment index in terms of the strength of associations with obesity-related cancer mortality rates.

## Methods

### Population and scale

This observational, cross-sectional study included all counties in 50 states and the District of Columbia, drawing on county-level obesity-related cancer mortality rates (2015–2020) from the CDC [41]. County-level covariates such as demographic and economic data were sourced from publicly available data sets (Additional file 1: Table S2). We constructed county-level location-based and activity-based indices of the retail food environment (RFE) using InfoGroup Historical Data and SafeGraph’s Core Places and Patterns datasets. Our final, main analytical sample included 2925 counties with complete information on RFE measures, obesity-related cancer mortality rates, and other demographic and economic variables. All data used in this study were de-identified and publicly available. We followed the Strengthening the Reporting of Observational Studies in Epidemiology (STROBE) reporting guideline.

### Measures

#### Outcome

The main outcome was the 5-year average county-level obesity-related cancer mortality rates 2015–2020. Following previous literature [5, 18], we used the age-adjusted mortality rate (per 100,000 population) from the CDC that consisted of 13 obesity-related cancer types (see Additional file 1: Table S1). As a robustness check, we also considered the mortality rate for each year separately.

#### Retail food environment (RFE) measures

We constructed two county-level retail food environment (RFE) measures. First, following a similar approach to the modified retail food environment index (mRFEI) [15], we constructed a location-based index with up-to-date data (2018–2019) by calculating the percentage of healthy food retailers out of all qualified food retailers (*N* = 471,725). This update is necessary because the existing mRFEI is based on business listings of 2008–2009. The location-based index defines healthy and less healthy food retailers based on the 2012 North American Industry Classification System (NAICS) codes. Healthy food retailers include supermarkets and larger grocery stores (NAICS 445110), warehouse clubs (NAICS 452910), and fruit and vegetable markets (NAICS 445230). Less healthy food retailers include limited-service restaurants (NAICS 722513) and convenience stores (NAICS 445120). We also complemented other limited-service restaurants by including food carry-out (SIC code 5812–06) and pizza restaurants (SIC code 5812–22). The data was sourced from InfoGroup Historical Data (2018–2019) [42], which provides a comprehensive list of business establishments in the US. The number of included food establishments under each NAICS category aligned well with the total number of businesses listed in the 2021 NAICS association statistics [34].

Second, we constructed a county-level activity-based index based on residents’ visits to food retailers in 2018 and 2019 using SafeGraph’s Core Places and Patterns datasets [43]. In contrast to the location-based index, the activity-based index estimates the percentage of visits to healthy food retailers out of total visits to both healthy and less healthy food retailers for people living in the same county. The definitions of healthy and less healthy food retailers follow the ones used in the location-based index. The visits were based on anonymized GPS-tracking data aggregated from approximately 10% of all GPS-enabled mobile devices in the US. SafeGraph determined a device’s “home” by analyzing nighttime data (6:00 PM to 7:00 AM) over 6 weeks and assigning it to a Geohash-7 grid (153 × 153 m), which was then mapped to census block groups, census tracts, and counties. This index was formulated using 94,256,870 aggregated visit records to 359,365 food retailers in 2018 and 2019 across the US, where each record indicated the destination store, origin (the visitor’s home census tract), and the number of visits in each year. This activity-based index was previously validated, and additional details about this activity-based index can be found in our previous work [34]. Both the location-based index and activity-based index are expressed as proportions in the subsequent analyses (ranging 0–1, where smaller number indicates a less healthy food environment).

#### Covariates

To capture relevant demographic, economic, and social characteristics of different counties, we considered a series of covariates that have been widely recognized in the literature as influencing obesity-related cancer mortality [18, 43–47], including racial and ethnic composition, percentage of senior population (aged 65 +), median household income, poverty rate, urbanity, education level, and food desert status. In Additional file 1: Table S2, we provide details of how these variables were measured and the source of the data. The social vulnerability index (SVI) data from the US Centers for Disease Control and Prevention [48] were also extracted as additional covariates as validated measures of community vulnerability to natural or human-caused stressors [49–51]. It uses 16 variables from the 5-year American Community Survey (ACS) to identify communities that may require support before, during, or after disasters. These variables are grouped into four themes: socioeconomic status, household composition and disability, minority status and language, and housing and transportation. The percentile ranking of each theme for each county (ranging from 0 to 1, whereas 1 means the most socially vulnerable) was included as additional covariates for further analyses.

### Statistical analysis

We conducted descriptive analyses, comparing two RFE measures and a range of demographic, economic, and social characteristics, stratified by whether the obesity-related cancer mortality rates were above or below the national median. Statistical comparisons were made using the Wilcoxon rank sum tests. The spatial distribution of the two RFE measures was also visualized to explore potential differences in the patterns they capture.

To investigate the associations between each RFE measure and obesity-related cancer mortality, we first examined the bivariate relationship using Pearson’s correlation to assess the linear trends. Next, we applied multivariable linear regression models, adjusting for demographic, economic, and social characteristics of each county. Furthermore, we dichotomized the obesity-related cancer mortality based on the national median (i.e., high-risk areas vs. low-risk areas), and conducted binary logistic regression analyses to assess its association with each RFE measure in separate analyses (with the same set of covariates as above). Both RFE measures were standardized before all regression analyses so that their coefficients can be compared.

We employed a series of additional analyses as a robustness check on our findings. First, we conducted the analyses using both the 5-year average mortality rate and the annual mortality rates from 2015 to 2020 as the outcome. We also assessed how results may change by adding SVIs as additional control variables to adjust for community-level social, economic, and demographic vulnerabilities. While the main model already includes a series of demographic, economic, and social characteristics of each county that substantially overlap with the SVI—they are not identical. Including SVI in the sensitivity analyses allowed us to assess the robustness of our findings while avoiding potential over-adjustment in the main model. In addition, we accounted for possible spatial autocorrelations in a series of spatial regression models. Generalized additive models (GAMs) were also employed to capture potential nonlinear effects between the obesity-related cancer mortality rates and each RFE measure. Lastly, we performed exploratory analyses to investigate potential heterogeneity in the associations between each RFE measure and obesity-related cancer mortality by adding interaction terms between each RFE measure and covariates, assessing whether the association between RFE and cancer mortality rates varies across different community contexts. For the covariates with the strongest interaction effects, we further performed stratified analyses (split at the median of the covariate) using separate multivariable linear regression models with the same specifications as the main analysis. All statistical tests were two-sided, with a significance level set at 5%. Statistical analyses were performed using R (version 4.4.1, R Foundation for Statistical Computing).

## Results

In Table 1, we compare the two RFE measures between counties with obesity-related cancer mortality rates above and below the national median (60.2 per 100,000 population). There was no statistically significant difference in the location-based index (i.e., the new mRFEI) between the two groups (median [IQR]: 0.21 [0.16, 0.29] for low-risk counties and 0.21 [0.16, 0.30] for high-risk counties, *P* = 0.8). However, counties with below-median mortality rates had a significantly higher activity-based index compared to those with above-median rates (low-risk counties: 0.25 [0.20, 0.31]; high-risk counties: 0.22 [0.17, 0.26], *P* < 0.001).

**Table 1:** RFE measures, demographic, economic, and social characteristics for counties with above (high-risk) and below (low-risk) national median obesity-related cancer mortality rates

Variable	Below median, *N* = 1460	Above median, *N* = 1465	*P* value
**Location-based food environment index**	0.21 (0.16, 0.29)	0.21 (0.16, 0.30)	0.8
**Activity-based food environment index**	0.25 (0.20, 0.31)	0.22 (0.17, 0.26)	< 0.001
**Population density (people per km**^**2**^**)**	21 (9, 69)	17 (8, 36)	< 0.001
**% White**	89 (79, 95)	89 (71, 95)	0.2
**% Black**	2 (1, 9)	3 (1, 15)	< 0.001
**% Hispanic**	5 (3, 11)	3 (2, 7)	< 0.001
**% Senior (over 65 +)**	17.7 (14.9, 20.7)	18.0 (15.8, 20.3)	0.11
**% Urban**	0.0 (0.0, 68.0)	0.0 (0.0, 0.0)	< 0.001
**% Suburban**	20.0 (0.0, 50.0)	17.0 (0.0, 50.0)	0.11
**% Poverty**	13.5 (10.1, 17.2)	16.3 (12.7, 20.8)	< 0.001
**Median household income (USD)**	53,306 (45,369, 62,497)	46,610 (40,383, 53,354)	< 0.001
**% No high school diploma**	10.9 (7.8, 15.4)	13.6 (10.0, 18.7)	< 0.001
**Percentile ranking of SVI (theme 1: socioeconomic)**	0.40 (0.19, 0.66)	0.62 (0.38, 0.82)	< 0.001
**Percentile ranking of SVI (theme 2: household composition and disability)**	0.38 (0.17, 0.64)	0.62 (0.39, 0.82)	< 0.001
**Percentile ranking of SVI (theme 3: minority status and language)**	0.54 (0.29, 0.77)	0.47 (0.22, 0.72)	< 0.001
**Percentile ranking of SVI (theme 4: housing type and transportation)**	0.47 (0.24, 0.72)	0.57 (0.31, 0.80)	< 0.001
**Food desert proportion (population-weighted)**	0.19 (0.00, 0.35)	0.25 (0.06, 0.41)	< 0.001

Median (IQR) are reported for all variables. *P* values are computed based on Wilcoxon rank sum tests to compare counties below and above national median age-adjusted obesity-related cancer mortality rates 2015–2020 (60.2 per 100,000 population). See Additional file 1: Table S2 for more details regarding definitions and data sources for all variables listed here

Additionally, significant differences were observed in other demographic, economic, and social characteristics between the high-risk and low-risk counties (Table 1). High-risk counties had higher proportions of African American residents (low-risk: 2% [1%, 9%], high-risk: 3% [1%, 15%]; *P* < 0.001), higher poverty rates (low-risk: 13.5% [10.1%, 17.2%], high-risk: 16.3% [12.7%, 20.8%], *P* < 0.001), lower median household income (low-risk: 53,306 [45,369, 62,497], high-risk: 46,610 [40,383, 53,354], *P* < 0.001), higher proportions of residents without a high school diploma (low-risk: 10.9% [7.8%, 15.4%], high-risk: 13.6% [10%, 18.7%], *P* < 0.001), and higher food desert prevalence (low-risk: 0.19 [0.00, 0.35]; high-risk: 0.25 [0.06, 0.41], *P* < 0.001).

**Fig. 1:**
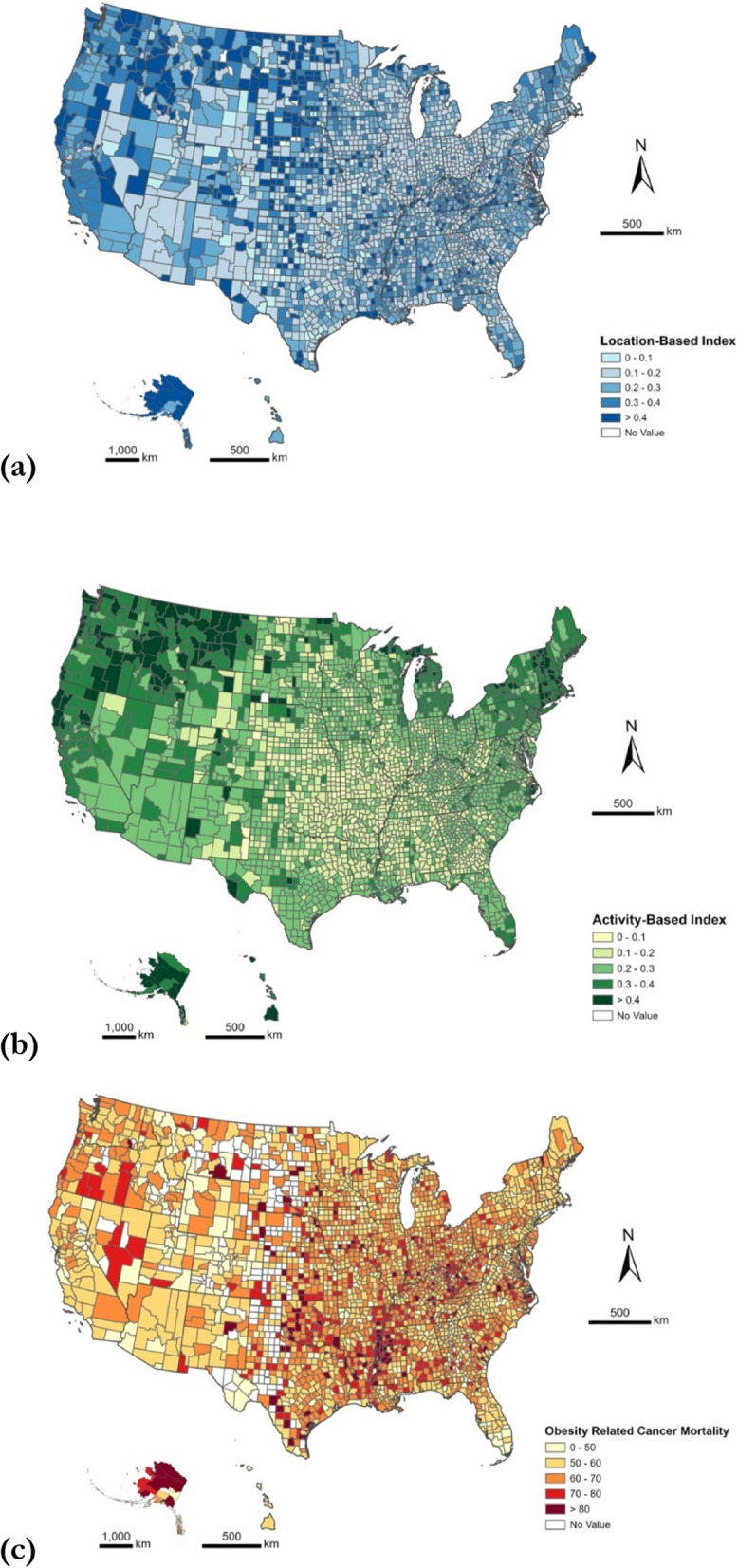
Spatial distribution of **a** location-based index, **b** activity-based index, and **c** the obesity-related cancer mortality rates (2015–2020)

**Fig. 2:**
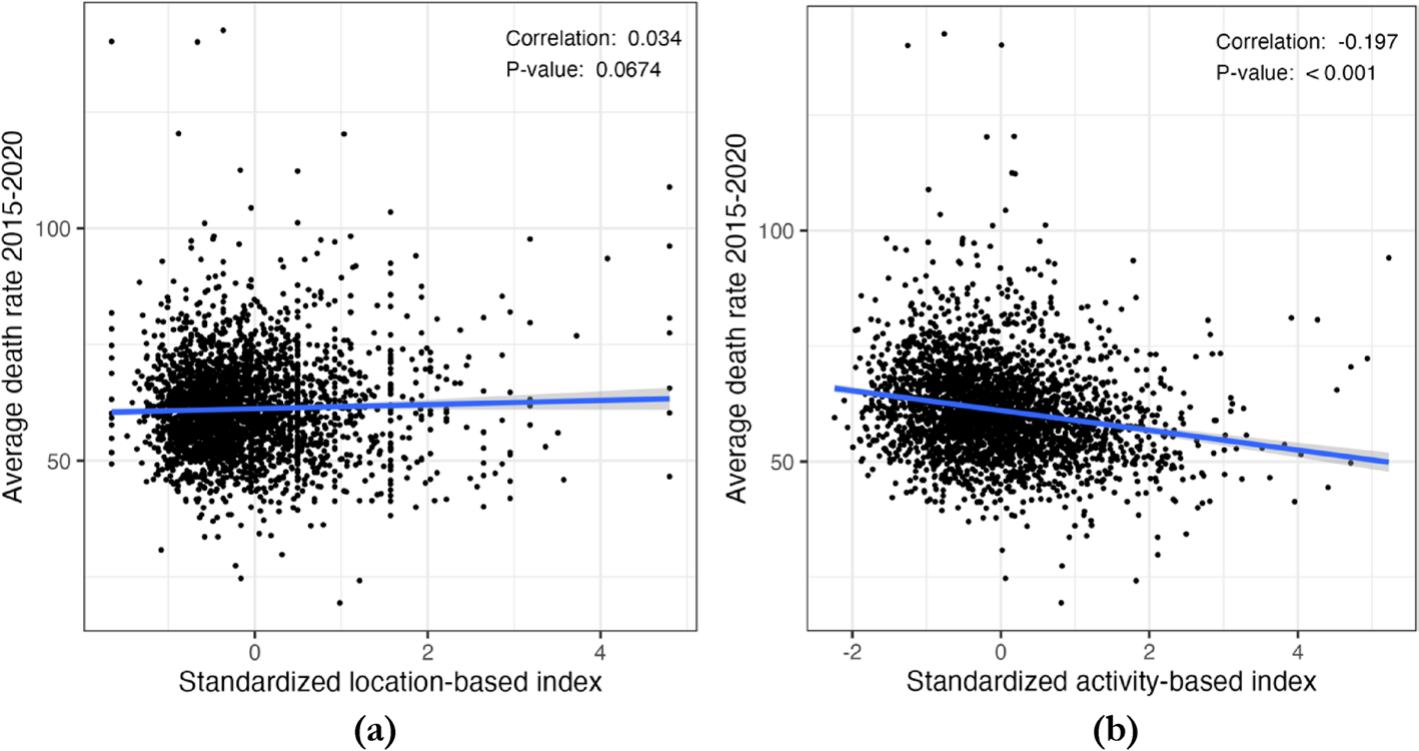
Scatterplots between standardized **a** location-based index and **b** activity-based index and 5-year average county-level obesity-related cancer mortality rates 2015–2020. Note. RFE measures are standardized in the scatterplots to make the two plots more comparable

Figure 1 depicts the spatial distributions of the two RFE measures and Fig. 2 shows the scatterplots of each RFE measure against the 5-year average obesity-related cancer mortality rates, with a fitted linear trend line. The correlation between the location-based index and the mortality rate is not statistically significant (correlation = 0.034, *P* = 0.067); however, there is a significant negative correlation between the activity-based index and mortality rate (correlation = − 0.197, *P* < 0.001), suggesting more visits to healthy food retailers are associated with a lower mortality rate.

**Fig. 3:**
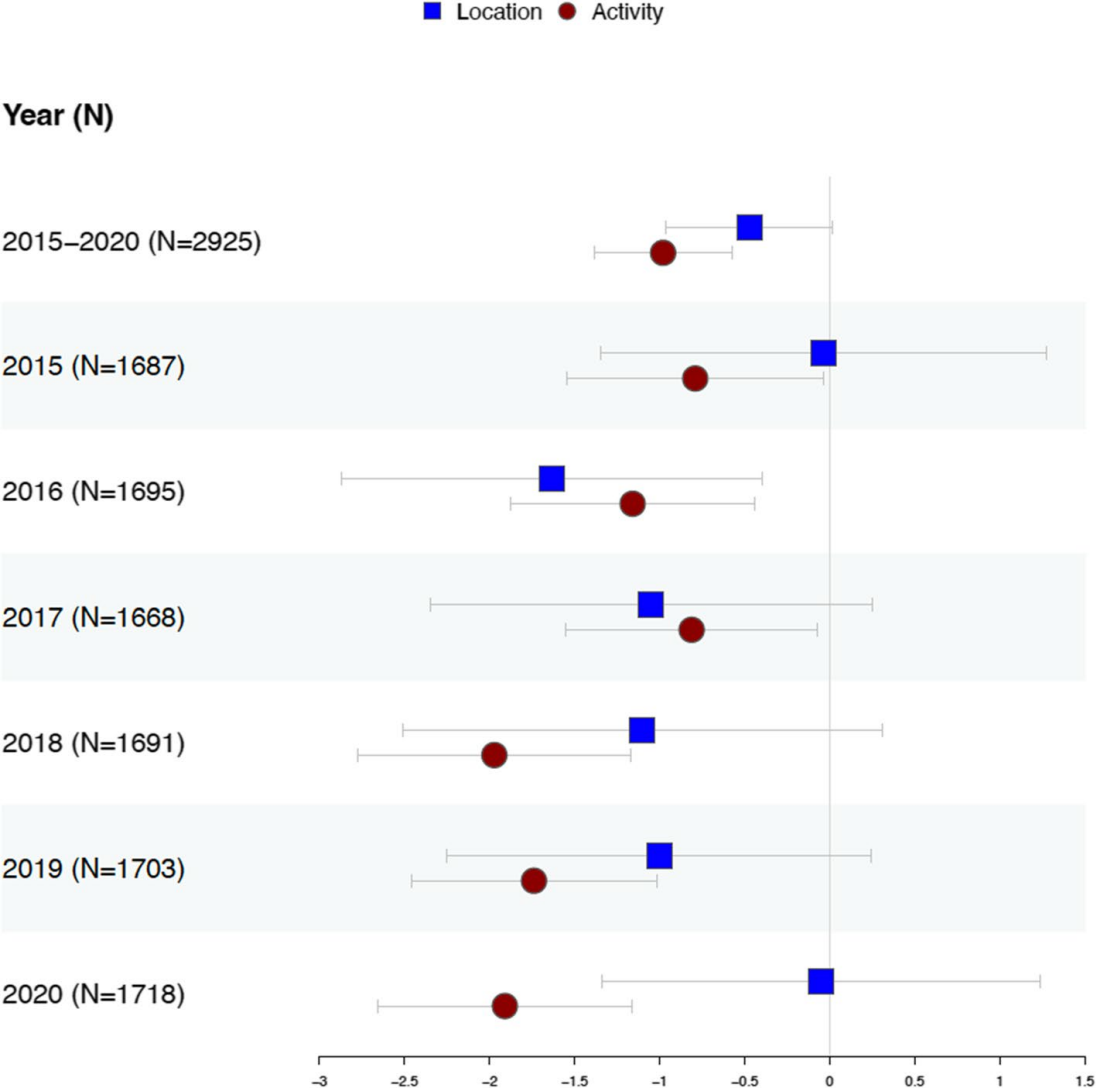
Forest plot of multivariable linear regression models predicting obesity-related cancer mortality rates using RFE measures (5-year average and annual mortality rates 2015–2020). Note. The forest plots present regression coefficients and 95% confidence intervals. RFE measures are standardized. All models are adjusted for the following control variables: population density, percentage of White, Black, and Hispanic populations, percentage of seniors, percentage of urban and suburban areas, percentage of the population living in poverty, median household income, percentage without a high school diploma, and the presence of food deserts. Detailed information on the construction of these variables is provided in Additional file 1: Table S2. Numerical results for these forest plots are available in Additional file 1: Table S3

**Fig. 4:**
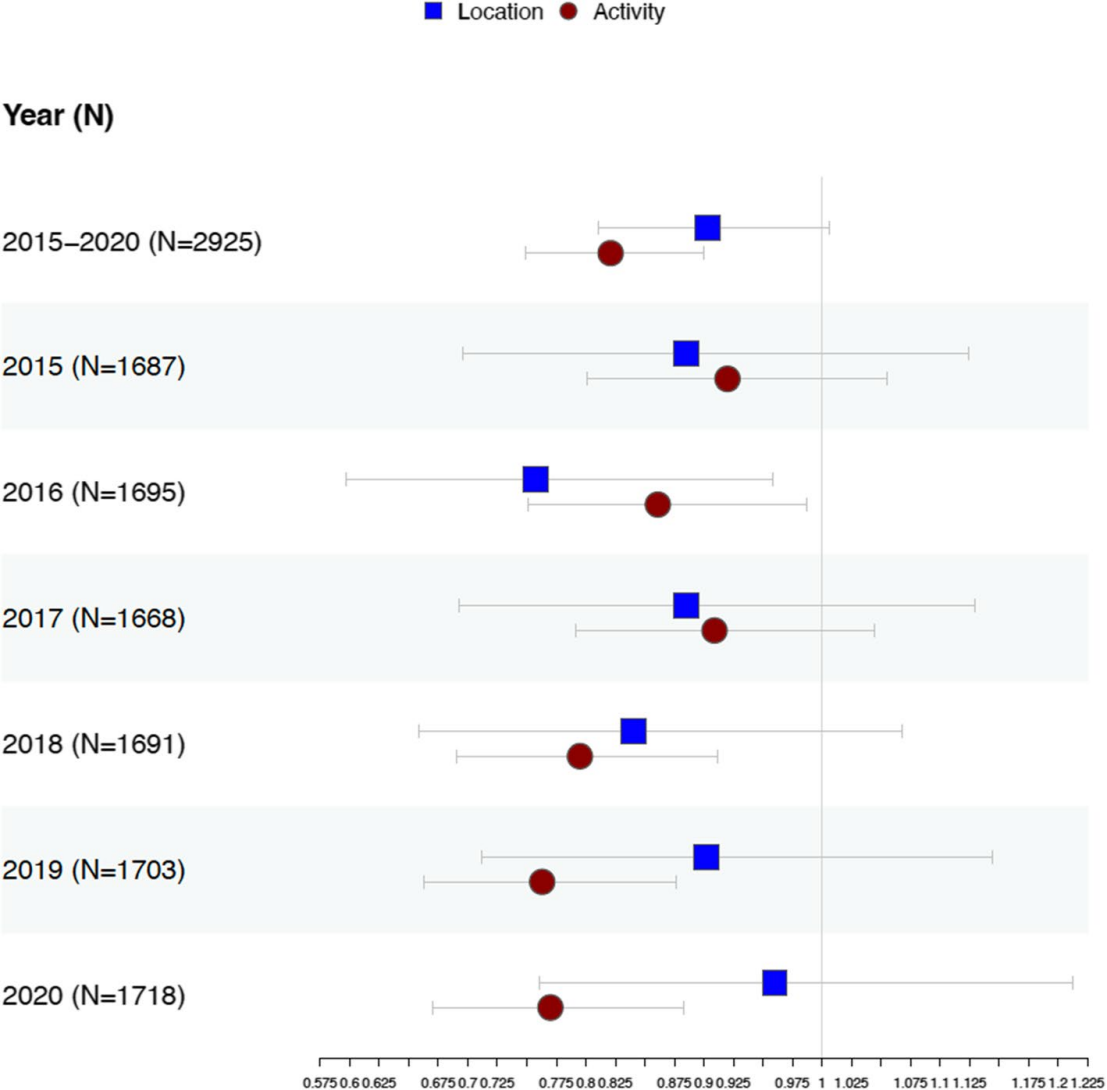
Forest plot of multivariable binary logistic regression models predicting obesity-related cancer mortality rates using RFE measures (5-year average and annual mortality rates 2015–2020). Note. The obesity-related cancer mortality was dichotomized at the national median. The national medians for the 5-year average, 2015, 2016, 2017, 2018, 2019, and 2020 are 60.2, 62.8, 61.1, 60.5, 60.1, 59.65, and 60.2 per 100,000 population, respectively. The forest plots present odds ratios and corresponding 95% confidence intervals. RFE measures are standardized. All models are adjusted for the following control variables: population density, percentage of White, Black, and Hispanic populations, percentage of seniors, percentage of urban and suburban areas, percentage of the population living in poverty, median household income, percentage without a high school diploma, and the presence of food deserts. Detailed information on the construction of these variables is provided in Additional file 1: Table S2. Numerical results for these forest plots are available in Additional file 1: Table S4

Figure 3 presents the results from the multivariable analyses of the two RFE measures, controlling for demographic, economic, and social characteristics. Both RFE measures are standardized so that the results can be compared. The patterns align with the bivariate correlations: the activity-based index consistently negatively associates with the mortality rate across both the 5-year average rate and the annual mortality rate (coefficient [95% CI]; 2015–2020: − 0.980 [− 1.385, − 0.575], *P* < 0.001; see Additional file 1: Table S3 for more details). In contrast, the location-based index shows minimal effect, with the 5-year average approaching significance (− 0.472 [− 0.961, 0.017], *P* = 0.058). Figure 4 presents the binary logistic regression results using dichotomized obesity-related cancer mortality rates (high- vs. low-risk counties) as the outcome and shows a similar pattern: the location-based index is significant only in 2016, while the activity-based index remains consistently significant across most years, except 2015 and 2017 (see Additional file 1: Table S4 for detailed results). Notably, one standard deviation increase in the activity-based index is associated with about 18% decrease in the odds of being a high-risk area (odds ratio [95% CI] for 5-year average: 0.821 [0.749, 0.900], *P* < 0.001), while one standard deviation increase in the location-based index is only associated with about 10% decrease in the odds of being a high-risk area (odds ratio [95% CI] for 5-year average: 0.903 [0.811, 1.006], *P* = 0.064).

Findings from the sensitivity analysis are presented in Additional file 1. Additional file 1: Tables S5 and S6 show results with SVIs added as additional covariates, and Additional file 1: Table S7 provides results from spatial regression models to account for the effect of spatial autocorrelation. Across all model specifications, a higher activity-based index remains consistently associated with a lower mortality rate (or lower odds of being a high-risk area), while the association between the location-based index and mortality rates is much weaker, if present at all. Finally, there are no significant non-linear relationships between each of the two RFE measures and the mortality rate, meaning that these non-linear effects do not distort the main results (Additional file 1: Fig. S1).

The exploratory analyses for heterogeneous associations are summarized in Additional file 1: Table S8 (moderation analyses) and Additional file 1: Table S9 (stratified results). Given the exploratory nature of these analyses, we highlight findings with the strongest statistical significance (*P* ≤ 0.001). For the location-based index, its association with obesity-related cancer mortality rates appears stronger in counties characterized by higher proportions of suburban areas (*P* < 0.001 for the interaction term) and minority populations (*P* = 0.001). Stratified analyses further revealed that the location-based index significantly predicted obesity-related cancer mortality only in communities with above-median suburban levels (− 1.53 [− 2.22, − 0.847], *P* < 0.001) and minority populations (− 1.34 [− 2.07, − 0.605], *P* < 0.001), while no significant association was observed in the below-median groups. In contrast, the activity-based index shows stronger associations with cancer mortality rates in counties with a higher proportion of Hispanic population (*P* = 0.001 for the interaction term) or lower vulnerability, as indicated by lower scores on SVI theme 1 (socioeconomic) (*P* < 0.001) and theme 2 (household composition and disability) (*P* < 0.001). Stratified analyses further indicated that the activity-based index significantly predicted mortality only in communities with above-median Hispanic population proportions (− 0.973 [− 1.55, − 0.395], *P* < 0.001) and in those with below-median vulnerability, as reflected by SVI theme 1 (− 1.41 [− 1.96, − 0.852], *P* < 0.001) and SVI theme 2 (− 1.33 [− 1.87, − 0.783], *P* < 0.001).

## Discussion

Our study contributes valuable insights into new approaches to measuring the food environment and examining their relationships with obesity-related cancer outcomes. By utilizing a county-level index derived from food retailer visits in the US, we demonstrate that incorporating human activities in the retail food environment can better predict obesity-related cancer mortality compared to using the location-based food environment index. Across models, we found that the effect of the activity-based index is about twice as strong as that of the location-based index in terms of the strength of association with obesity-related cancer mortality rates. This result aligns with past literature regarding the limitations and assumptions underlying location-based indices, which often fail to capture the complexities of the food environment. These complexities include not only the physical availability of food but also factors such as consumer behavior, mobility patterns, and social and cultural influences on food choices, all of which play a significant role in diet-related health outcomes [13, 32, 52].

The activity-based index offers a more nuanced perspective by capturing the frequency and patterns of visits to food retailers, which reflect the behaviors and choices of local residents. While visits do not necessarily equate to food procurement, they provide valuable insight into residents’ engagement with the local food environment that shapes dietary habits. This finding aligns with contemporary theories that conceptualize the local food environment as a dynamic, complex, multidimensional system [13, 53–55]. This perspective goes beyond the mere availability of food outlets and can incorporate non-spatial factors, such as the quality and price of food offerings and their alignment with the preferences and needs of local residents [56]. In contrast, location-based indices rely on assumptions about food exposure within a defined geographic area and overlook food behaviors like shopping at stores outside of local boundaries or selecting culturally relevant food sources. By integrating the behavioral component in the formation of the food environment index, the study captures critical human–environment interactions that influence long-term health outcomes, such as the obesity-related cancers as discussed in the paper, which are driven by a multitude of factors such as food availability, price, quality, store services, and marketing, as well as personal factors such as financial resources, environmental perceptions, transportation availability, and nutrition education [53, 56].

Our findings support the robust association between the activity-based index and obesity-related cancer mortality rates. This finding contributes to the ongoing debate on the relationship between the food environment and obesity-related health outcomes, where existing findings remain mixed [17–28]. It is also consistent with a previous study demonstrating that the activity-based index is a better predictor of the prevalence of cardiometabolic diseases than the location-based index [34]. Importantly, our findings remain significant even after adjusting for a wide range of demographic, economic, and social factors, as well as SVI scores and the food desert measure. This consistent association highlights the power of the activity-based index to capture behavioral dimensions not fully reflected in traditional food environment measures [32, 52]. However, we acknowledge that visits to food retailers are an imperfect proxy for food procurement activities, as they do not capture the types of food purchased or consumed. Nevertheless, visits provide valuable insights into residents’ interactions with the food environment. Store visits reflect key behavioral aspects of food exposure and can shape dietary patterns and health outcomes over the long term. The significant negative association we found between the activity-based index and obesity-related cancer mortality further supports the notion that where and how individuals engage with the food environment matters.

These findings have important policy implications. In recent decades, many policies aimed at improving diet-related health outcomes have focused on improving food access in areas deprived of healthy food stores (e.g., opening a healthy food retailer in a food desert). However, a systematic review revealed that the majority of these geographic access interventions showed no effects in improving local diets [57]. Our findings offer an alternative explanation for this policy deficiency: ameliorating areas of low food access does not necessarily enhance the quality of food procurement for local residents, as they may shop elsewhere. The findings further call for the need for future policy initiatives to go beyond geographic-based measures (e.g., USDA “low-income, low-access areas”) [14] and consider behavioral factors, such as the frequency and nature of visits to food outlets, which more accurately reflect the local food environment’s impact on diets. Integrating food retailer visits into evidence-based interventions could provide a more comprehensive approach to promoting healthy food consumption.

Furthermore, our exploratory analyses showed that the association between the activity-based index and obesity-related cancer mortality rates may vary by community context. Specifically, the stronger predictive power of the activity-based index in communities with higher proportions of Hispanic residents and higher socioeconomic status (SES) may reflect its ability to capture behavior-driven interactions with the food environment, in contrast to the location-based index. In Hispanic communities, traditional dietary practices and preferences, such as shopping at culturally specific markets or purchasing ethnic food, may lead to more procurement of healthy foods and better diet quality in Hispanic communities, aligning with the “Hispanic Paradox” observed in previous research [58–61], which suggests that cultural factors can moderate the effects of food access. Similarly, higher SES residents are more likely to have the health awareness to actively seek out healthier food retailers and the financial means to purchase healthy foods [55, 62, 63]. These findings highlight the new activity-based index’s potential in capturing nuanced, behavior-driven dynamics in the food environment but also point to its complexities. Future research should further investigate how sociodemographic, cultural, and economic factors shape these interactions to elucidate how humans interact with the food environment.

### Strengths and limitations

To the best of our knowledge, our findings are among the first to investigate the activity-based drivers of obesity-related cancers on a broad scale. Our investigation is based on valuable GPS-based longitudinal data (SafeGraph) on food visits across the US. Our findings are robust given all the sociodemographic and neighborhood characteristics controlled for analysis, as well as the various sensitivity analyses we conducted. Our study has several limitations: Firstly, being observational in nature, the study cannot infer causal relationships. Secondly, the translation of findings from an aggregate level to an individual level carries the risk of ecological fallacy, as the aggregated mobility data at the county level cannot capture unique food activity patterns at smaller geographic scales, such as blocks, households, and individuals. Another limitation is that the activity-based index focuses on visits to food outlets, which do not necessarily equate to actual food procurement or consumption. Future studies are needed to further explore how visits relate to other aspects of food behavior, such as purchasing decisions and dietary intake. Lastly, although SafeGraph sampling reflects a range of sociodemographic characteristics and aligns well with the census population, recent research suggests that specific groups, including Hispanic populations, low-income households, and individuals with lower educational attainment, may be underrepresented in this dataset [64]. This limitation is partly due to the reliance on location-based tracking systems, such as GPS data, which are subject to inherent biases in data collection. Factors like privacy concerns, differences in smartphone ownership and usage, and variability in app-based participation can result in incomplete or skewed representations of certain populations. Additionally, access to location-based data can be restricted by proprietary barriers, potentially limiting its availability for broader research applications. These technological and methodological constraints underscore the need for further studies to critically evaluate the biases in mobility data and to develop strategies for mitigating their impact. Addressing these issues is essential to improving the robustness and generalizability of activity-based indices in characterizing food environments and strengthening their health implications.

While activity-based food environment indices present a promising avenue for advancing policy frameworks, their application is not without challenges. The reliance on location-based tracking systems for data collection, such as GPS or mobile location services, introduces potential limitations, including incomplete or biased data, privacy concerns, technological disparities, and uneven data availability across geographic and socioeconomic contexts. To address these limitations and ensure broader applicability, future efforts should prioritize enhancing data quality, strengthening data ethics, and fully clarifying all data limitations in usage. Such efforts will be critical for building robust, inclusive datasets that accurately represent diverse populations and enable equitable, evidence-based policy interventions.

## Conclusions

In a US nationwide study, we show that a novel activity-based food environment index, representing food retailer visits, had significantly stronger associations with obesity-related cancer mortality rates than that of the location-based food environment index. This new index provides an alternative approach to identifying communities in need of healthy food provisioning. It has the potential to guide more effective policy actions and resource allocations aimed at reducing obesity-related diseases and mortality.

## Supplementary Information

Additional file 1: Table S1. ICD-10 codes for identification of obesity-related cancers. Table S2. Covariates (county-level) definitions and data sources. Table S3. Multivariable linear regression models predicting obesity-related cancer mortality rates using standardized RFE measures (5-year average and annual mortality rates from 2015 to 2020). Table S4. Multivariable binary logistic regression models predicting obesity-related cancer mortality rates using standardized RFE measures (5-year average and annual mortality rates from 2015 to 2020). Table S5. Multivariable linear regression models predicting obesity-related cancer mortality rates using standardized RFE measures (5-year average and annual mortality rates from 2015 to 2020), with SVI as additional covariates. Table S6. Multivariable binary logistic regression models predicting obesity-related cancer mortality rates using standardized RFE measures (5-year average and annual mortality rates from 2015 to 2020), with SVI as additional covariates. Table S7. Spatial regression models (spatial lag and spatial error models) predicting obesity-related cancer mortality rates using standardized RFE measures (5-year average and annual mortality rates from 2015 to 2020). Table S8. Moderating effects of community context on the association between obesity-related cancer mortality rates (5-year average rates from 2015 to 2020) and standardized RFE measures using multivariable linear regression models. Table S9. Stratified results between obesity-related cancer mortality rates (5-year average rates from 2015 to 2020) and standardized food environment measures using multivariable linear regression models. Fig. S1. Generalized additive models predicting 5-year average obesity-related cancer mortality rates using each RFE measure.

## Data Availability

The datasets used and analyzed at the county level during the current study are available from the corresponding author on a reasonable request.

## Abbreviations

CDC: Centers for Disease Control and Prevention
GAMs: Generalized additive models
GPS: Global Positioning System
IARC: International Agency for Research on Cancer
mRFEI: Modified retail food environment index
NAICS: North American Industry Classification System
RFE: Retail food environment
SVI: Social vulnerability index
USDA: United States Department of Agriculture
