# Supplementary material for: Association of activity-based food environment index with obesity-related cancer mortality in the US
Source: BMC Med. 2025 Mar 20;23:167. doi: 10.1186/s12916-025-03967-6 (PMC11927273; doi:10.1186/s12916-025-03967-6)
Supplement: Supplementary file 1 — Additional file 1: Table S1. ICD-10 codes for identification of obesity-related cancers. Table S2. Covariates (county-level) definitions and data sources. Table S3. Multivariable linear regression models predicting obesity-related cancer mortality rates using standardized RFE measures (5-year average and annual mortality rates from 2015 to 2020). Table S4. Multivariable binary logistic regression models predicting obesity-related cancer mortality rates using standardized RFE measures (5-year average and annual mortality rates from 2015 to 2020). Table S5. Multivariable linear regression models predicting obesity-related cancer mortality rates using standardized RFE measures (5-year average and annual mortality rates from 2015 to 2020), with SVI as additional covariates. Table S6. Multivariable binary logistic regression models predicting obesity-related cancer mortality rates using standardized RFE measures (5-year average and annual mortality rates from 2015 to 2020), with SVI as additional covariates. Table S7. Spatial regression models (spatial lag and spatial error models) predicting obesity-related cancer mortality rates using standardized RFE measures (5-year average and annual mortality rates from 2015 to 2020). Table S8. Moderating effects of community context on the association between obesity-related cancer mortality rates (5-year average rates from 2015 to 2020) and standardized RFE measures using multivariable linear regression models. Table S9. Stratified results between obesity-related cancer mortality rates (5-year average rates from 2015 to 2020) and standardized food environment measures using multivariable linear regression models. Fig. S1. Generalized additive models predicting 5-year average obesity-related cancer mortality rates using each RFE measure. [file 12916_2025_3967_MOESM1_ESM.docx]

**Additional file 1**

**Table S1.** ICD-10 codes for identification of obesity-related cancers

| **ICD-10 Code** | **Definition and/or Technical Information** |
| --- | --- |
| C15.9 | Esophagus, unspecified – Malignant neoplasms |
| C16.0 | Cardia – Malignant neoplasms |
| C18.9 | Colon, unspecified – Malignant neoplasms |
| C22.0 | Liver cell carcinoma – Malignant neoplasms |
| C23.0 | Malignant neoplasm of gallbladder |
| C25.9 | Pancreas, unspecified – Malignant neoplasms |
| C32.9 | Larynx, unspecified – Malignant neoplasms |
| C50.9 | Breast, unspecified – Malignant neoplasms |
| C54.9 | Corpus uteri, unspecified – Malignant neoplasms |
| C56.0 | Malignant neoplasm of ovary |
| C64.0 | Malignant neoplasm of kidney, except renal pelvis |
| C73.0 | Malignant neoplasm of thyroid gland |
| C90.0 | Multiple myeloma – Malignant neoplasms |

**Table S2.** Covariates (county-level) definitions and data sources.

| **Measure** | **Definition** | **Data source** |
| --- | --- | --- |
| % white | Percentage of population with race identified as white alone. | American Community Survey (ACS), 2018 5-year average |
| % black | Percentage of population with race identified as Black or African American alone. | ACS, 2018 5-year average |
| % hispanic | Percentage of population with ethnicity identified as of Hispanic or Latinx origin. | ACS, 2018 5-year average |
| % senior (over 65 years old) | Percentage of population over 65. | ACS, 2018 5-year average |
| % Urban | Percentage census tracts in the county classified as Urban using RUCA codes. | Paykin, Susan & Menghaney, Moksha & Lin, Qinyun & Kolak, Marynia. (2021). Rural, Suburban, Urban Classification for Small Area Analysis. 10.13140/RG.2.2.25148.16009. |
| % Suburban | Percentage census tracts in the county classified as Suburban using RUCA codes. | Paykin, Susan & Menghaney, Moksha & Lin, Qinyun & Kolak, Marynia. (2021). Rural, Suburban, Urban Classification for Small Area Analysis. 10.13140/RG.2.2.25148.16009. |
| % poverty | Number of individuals earning below the poverty income threshold as a percentage of the total population. | ACS, 2018 5-year average |
| Median household income | Median household income (in USD) | ACS, 2018 5-year average |
| % no high school diploma | Percentage of population 25 years and over, less than a high school degree | ACS, 2018 5-year average |
| SVI (THEME1) | SVI Ranking, Theme 1: socioeconomic. | CDC Social Vulnerability Index 2018 |
| SVI (THEME2) | SVI Ranking, Theme 2: Household Composition & Disability. | CDC Social Vulnerability Index 2018 |
| SVI (THEME3) | SVI Ranking, Theme 3: Minority Status & Language. | CDC Social Vulnerability Index 2018 |
| SVI (THEME4) | SVI Ranking, Theme 4: Housing Type & Transportation. | CDC Social Vulnerability Index 2018 |
| Food Desert | Binary indicator defined by low income and low access measured at ½ (urban) and 10 miles (rural) at the census tract level, then aggregated to the county level weighted by population. | USDA |

**Table S3.** Multivariable linear regression models predicting obesity-related cancer mortality rates using standardized RFE measures (five-year average and annual mortality rates from 2015 to 2020).

|  | **Location-based index** | | **Activity-based index** | |
| --- | --- | --- | --- | --- |
|  | **Coefficient (95% CI)** | **p-value** | **Coefficient (95% CI)** | **p-value** |
| 2015–2020 (N = 2925) | -0.472 (-0.961, 0.017) | 0.058 | -0.980  (-1.385, -0.575) | < 0.001 |
| 2015 (N = 1687) | -0.036  (-1.349, 1.277) | > 0.9 | -0.791 (-1.545, -0.037) | 0.04 |
| 2016 (N = 1695) | -1.632  (-2.869, -0.395) | 0.010 | -1.159 (-1.877, -0.442) | 0.002 |
| 2017 (N = 1668) | -1.049 (-2.351, 0.254) | 0.110 | -0.812 (-1.550, -0.074) | 0.031 |
| 2018 (N = 1691) | -1.101 (-2.507, 0.306) | 0.130 | -1.972 (-2.771, -1.173) | < 0.001 |
| 2019 (N = 1703) | -1.002 (-2.251, 0.246) | 0.120 | -1.739  (-2.461, -1.016) | < 0.001 |
| 2020 (N = 1718) | -0.054 (-1.343, 1.235) | > 0.9 | -1.910 (-2.656, -1.163) | < 0.001 |

***Note***. This table presents the numeric results for Figure 3.

**Table S4.** Multivariable binary logistic regression models predicting obesity-related cancer mortality rates using standardized RFE measures (five-year average and annual mortality rates from 2015 to 2020).

|  | **Location-based index** | | **Activity-based index** | |
| --- | --- | --- | --- | --- |
|  | **Odds ratio (95% CI)** | **p-value** | **Odds ratio (95% CI)** | **p-value** |
| 2015–2020 (N = 2925) | 0.903 (0.811, 1.006) | 0.064 | 0.821 (0.749, 0.900) | < 0.001 |
| 2015 (N = 1687) | 0.885 (0.696, 1.124) | 0.300 | 0.920 (0.801, 1.055) | 0.200 |
| 2016 (N = 1695) | 0.757 (0.597, 0.958) | 0.021 | 0.861 (0.751, 0.987) | 0.032 |
| 2017 (N = 1668) | 0.885 (0.693, 1.130) | 0.300 | 0.909 (0.792, 1.044) | 0.200 |
| 2018 (N = 1691) | 0.840 (0.659, 1.068) | 0.200 | 0.795 (0.691, 0.912) | 0.001 |
| 2019 (N = 1703) | 0.902 (0.712, 1.144) | 0.400 | 0.763 (0.663, 0.877) | < 0.001 |
| 2020 (N = 1718) | 0.960  (0.761, 1.212) | 0.700 | 0.770 (0.670, 0.883) | < 0.001 |

***Note***. This table presents the numeric results for Figure 4.

**Table S5.** Multivariable linear regression models predicting obesity-related cancer mortality rates using standardized RFE measures (five-year average and annual mortality rates from 2015 to 2020), with SVI as additional covariates.

|  | **Location-based index** | | **Activity-based index** | |
| --- | --- | --- | --- | --- |
|  | **Coefficient (95% CI)** | **p-value** | **Coefficient (95% CI)** | **p-value** |
| 2015–2020 (N = 2925) | -0.342 (-0.823, 0.140) | 0.2 | -0.693  (-1.098, -0.288) | < 0.001 |
| 2015 (N = 1687) | 0.387  (-0.918, 1.693) | 0.6 | -0.554 (-1.315, 0.207) | 0.2 |
| 2016 (N = 1695) | -1.403  (-2.637, -0.169) | 0.026 | -1.096 (-1.822, -0.370) | 0.003 |
| 2017 (N = 1668) | -0.706 (-2.004, 0.592) | 0.300 | -0.607 (-1.351, 0.137) | 0.110 |
| 2018 (N = 1691) | -0.778 (-2.177, 0.621) | 0.300 | -1.762 (-2.568, -0.956) | < 0.001 |
| 2019 (N = 1703) | -0.560 (-1.796, 0.676) | 0.400 | -1.415  (-2.142, -0.688) | < 0.001 |
| 2020 (N = 1718) | -0.469 (-0.812, 1.749) | 0.5 | -1.540 (-2.291, -0.789) | < 0.001 |

**Table S6.**Multivariable binary logistic regression models predicting obesity-related cancer mortality rates using standardized RFE measures (five-year average and annual mortality rates from 2015 to 2020), with SVI as additional covariates.

|  | **Location-based index** | | **Activity-based index** | |
| --- | --- | --- | --- | --- |
|  | **Odds ratio (95% CI)** | **p-value** | **Odds ratio (95% CI)** | **p-value** |
| 2015–2020 (N = 2925) | 0.936 (0.838, 1.045) | 0.2 | 0.859 (0.781, 0.944) | 0.002 |
| 2015 (N = 1687) | 0.941 (0.738, 1.198) | 0.600 | 0.954 (0.828, 1.099) | 0.500 |
| 2016 (N = 1695) | 0.791 (0.622, 1.003) | 0.054 | 0.883 (0.767, 1.015) | 0.081 |
| 2017 (N = 1668) | 0.931 (0.727, 1.191) | 0.600 | 0.937 (0.813, 1.079) | 0.400 |
| 2018 (N = 1691) | 0.871 (0.682, 1.110) | 0.300 | 0.822 (0.713, 0.947) | 0.007 |
| 2019 (N = 1703) | 0.963 (0.758, 1.226) | 0.800 | 0.797 (0.690, 0.920) | 0.002 |
| 2020 (N = 1718) | 1.040 (0.821, 1.319) | 0.700 | 0.814 (0.707, 0.937) | 0.004 |

**Table S7.** Spatial regression models (spatial lag and spatial error models) predicting obesity-related cancer mortality rates using standardized RFE measures (five-year average and annual mortality rates from 2015 to 2020).

|  |  | **Location-based index** | | **Activity-based index** | |
| --- | --- | --- | --- | --- | --- |
|  |  | **Coefficient (SE)** | **p-value** | **Coefficient (SE)** | **p-value** |
| 2015–2020 (N = 2925) | SLM | -0.309 (0.242) | 0.201 | -0.624 (0.203) | 0.002 |
|  | SEM | -0.269 (0.249) | 0.279 | -0.792 (0.241) | 0.001 |
| 2015 (N = 1687) | SLM | -0.0242 (0.667) | 0.971 | -0.751 (0.384) | 0.05 |
|  | SEM | 0.139 (0.690) | 0.840 | -0.632 (0.412) | 0.126 |
| 2016 (N = 1695) | SLM | -1.617 (0.628) | 0.010 | -1.145 (0.365) | 0.002 |
|  | SEM | -1.330 (0.650) | 0.041 | -1.081 (0.396) | 0.006 |
| 2017 (N = 1668) | SLM | -1.044 (0.660) | 0.113 | -0.754 (0.374) | 0.044 |
|  | SEM | -0.877 (0.687) | 0.202 | -0.730 (0.409) | 0.074 |
| 2018 (N = 1691) | SLM | -1.018 (0.712) | 0.153 | -1.866 (0.406) | < 0.001 |
|  | SEM | -0.510 (0.745) | 0.494 | -1.939 (0.450) | < 0.001 |
| 2019 (N = 1703) | SLM | -0.969 (0.633) | 0.126 | -1.669 (0.367) | < 0.001 |
|  | SEM | -0.592 (0.655) | 0.367 | -1.575 (0.398) | < 0.001 |
| 2020 (N = 1718) | SLM | 0.0009 (0.651) | 0.999 | -1.792 (0.378) | < 0.001 |
|  | SEM | 0.468 (0.678) | 0.490 | -1.757 (0.411) | < 0.001 |

***Note****.* SLMs are spatial lag models and SEMs are spatial error models. All models adjust for the following control variables: population density, percentage of White, Black, and Hispanic residents, percentage of seniors, percentage of urban and suburban areas, percentage of the population living in poverty, median household income, percentage without a high school diploma, and the presence of food deserts. Detailed information on the construction of these variables is provided in Table S2. First-order queen contiguity weights are used in all models.

**Table S8**. Moderating effects of community context on the association between obesity-related cancer mortality rates (five-year average rates from 2015 to 2020) and standardized RFE measures using multivariable linear regression models.

|  | **Location-based index** | | **Activity-based index** | |
| --- | --- | --- | --- | --- |
|  | **Coefficient (95% CI)** | **p-value** | **Coefficient (95% CI)** | **p-value** |
| **Population density** (people per km^2^) | -0.001 (-0.002, 0) | 0.050 | 0 (-0.001, 0.001) | 0.591 |
| **% white** | 0.006 (-0.017, 0.029) | 0.608 | -0.019 (-0.039, 0.001) | 0.064 |
| **% black** | -0.046 (-0.077, -0.015) | 0.004 | 0.008 (-0.023, 0.04) | 0.598 |
| **% hispanic** | -0.044 (-0.076, -0.011) | 0.009 | -0.057 (-0.091, -0.023) | 0.001 |
| **% senior (over 65 years old)** | 0.084 (-0.008, 0.176) | 0.072 | 0.003 (-0.069, 0.074) | 0.938 |
| **% Urban** | -2.309 (-4.68, 0.062) | 0.056 | 0.559 (-0.726, 1.844) | 0.393 |
| **% Suburban** | -3.494 (-4.822, -2.166) | <0.001 | -0.478 (-1.751, 0.796) | 0.462 |
| **% poverty** | 0.033 (-0.03, 0.096) | 0.306 | 0.069 (0.008, 0.129) | 0.027 |
| **Median household income** (USD) | 0.000 (-0.000, 0.000) | 0.064 | 0.000 (-0.000, 0.000) | 0.143 |
| **% no high school diploma** | -0.032 (-0.107, 0.043) | 0.402 | 0.088 (0.024, 0.152) | 0.007 |
| **Percentile ranking of SVI (THEME1: Socioeconomic)** | -0.399 (-1.882, 1.083) | 0.597 | 2.707 (1.402, 4.013) | <0.001 |
| **Percentile ranking of SVI (THEME2: Household Composition & Disability)** | 1.473 (-0.128, 3.075) | 0.071 | 2.536 (1.252, 3.819) | <0.001 |
| **Percentile ranking of SVI (THEME3: Minority Status & Language)** | -2.62 (-4.121, -1.12) | 0.001 | -0.311 (-1.65, 1.028) | 0.649 |
| **Percentile ranking of SVI (THEME4: Housing Type & Transportation)** | -1.123 (-2.634, 0.388) | 0.145 | 1.172 (-0.115, 2.459) | 0.074 |
| **Food Desert** **proportion (weighted by population)** | 1.219 (-0.239, 2.676) | 0.101 | 1.264 (-0.154, 2.682) | 0.081 |

Note. The table reports the coefficient, 95% confidence interval, together with P-value for the interaction term between each standardized RFE measure and each sociodemographic variable. Each interaction term is tested in a separate model, with all other covariates (including SVI) included in the model.

**Table S9**. Stratified results between obesity-related cancer mortality rates (five-year average rates from 2015 to 2020) and standardized food environment measures using multivariable linear regression models.

|  | **Stratify** | **Coefficient (95% CI)** | **P-value** |
| --- | --- | --- | --- |
| **Location-based index** | | | |
| % Suburban | Low | 0.545 (-0.149, 1.24) | 0.123 |
|  | High | -1.53 (-2.22, -0.847) | <0.001 |
| Percentile ranking of SVI (Theme 3: Minority Status & Language) | Low | 0.340 (-0.317, 0.998) | 0.310 |
|  | High | -1.34 (-2.07, -0.605) | <0.001 |
| **Activity-based index** | | | |
| % Hispanic | Low | -0.559 (-1.13, 0.0137) | 0.056 |
|  | High | -0.973 (-1.55, -0.395) | <0.001 |
| Percentile ranking of SVI (Theme 1: Socioeconomic) | Low | -1.41 (-1.96, -0.852) | <0.001 |
|  | High | 0.137 (-0.457, 0.732) | 0.651 |
| Percentile ranking of SVI (Theme 2: Household Composition & Disability) | Low | -1.33 (-1.87, -0.783) | <0.001 |
|  | High | 0.020 (-0.587, 0.628) | 0.948 |

Note. The table reports the coefficient, 95% confidence interval, and the P-value for the standardized food environment measure. By stratified results, we mean the data were split at the median and separate regression analyses were conducted. Median values for each covariate refer to: 4.1 for % Hispanic, 15 for % suburban, 0.4997 for SVI theme 1, 0.4998 for SVI theme 2, and 0.5 for SVI theme 3. All models are adjusted for the following control variables: population density, percentage of White, Black, and Hispanic populations, percentage of seniors, percentage of urban and suburban areas, percentage of the population living in poverty, median household income, percentage without a high school diploma, the presence of food deserts, and four SVI themes.

**Figure S1.** Generalized additive models predicting five-year average obesity-related cancer mortality rates using each RFE measure.

**
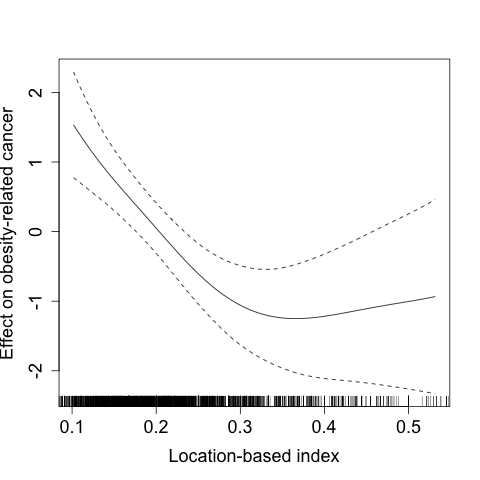

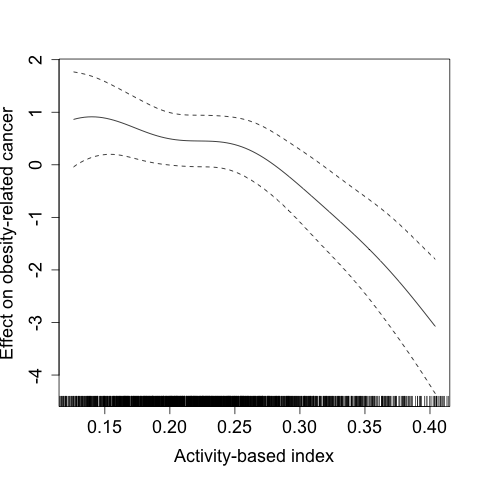

*Note*.** The plots are based on generalized additive models (GAMs) predicting five-year average obesity-related cancer mortality rates using each RFE measure. To minimize the influence of outliers, the GAM plots are based on data within the 5th to 95th percentile range. Both models adjust for the following control variables: population density, percentage of White, Black, and Hispanic residents, percentage of seniors, percentage of urban and suburban areas, percentage of the population living in poverty, median household income, percentage without a high school diploma, and the presence of food deserts. Detailed information on the construction of these variables is provided in Table S2. The results remain similar if SVI are added as additional covariates.
